# Post-exercise creatine kinase variability: a literature review

**DOI:** 10.11613/BM.2025.020502

**Published:** 2025-06-15

**Authors:** Vanja Radišić Biljak, Anja Lazić, Ana Nikler, Damir Pekas, Andrea Saračević, Nebojša Trajković

**Affiliations:** 1 Department of Medical Laboratory Diagnostics, University Hospital “Sveti Duh”, Zagreb, Croatia; 2 University of Zagreb, Faculty of Kinesiology, Zagreb, Croatia; 3 Faculty of sport and physical education, University of Niš, Niš, Serbia

**Keywords:** creatine kinase, sports medicine, exercise, variability

## Abstract

Creatine kinase (CK) activity has been generally considered as reliable blood marker for assessing muscle function, damage, and repair. However, the greatest challenge in the interpretation of CK activity remains the high variability in CK increase in relation to degrees of muscle cell damage or disturbance. Several known contributors to CK variability have been identified. The most important include the type of training, exercise intensity, gender differences, body composition, intra- and interindividual biological variability, as well as preanalytical and analytical considerations. Creatine kinase variability following different types of exercise reflects the complex interplay between exercise modality, intensity, individual physiology, and recovery strategies. High-intensity exercises, especially those involving eccentric muscle contractions, tend to produce more significant CK responses due to greater muscle fiber disruption. Gender differences in CK variability are pronounced, with men generally exhibiting higher CK activities following exercise compared to women. Creatine kinase variability is also closely linked to body composition, with muscle mass generally leading to higher CK activities post-exercise, while higher body fat may correlate with lower CK responses. Regarding preanalytical and analytical considerations, perhaps the greatest challenge in CK measurement is the limited sample stability, which should always be taken into consideration when analyzing CK activity in stored samples for research or clinical purposes. This review, through exploring all of the above-mentioned sources of CK variability, could facilitate the development of evidence-based practices for preventing overuse injuries, and promoting long-term athlete health and well-being.

## Graphical abstract

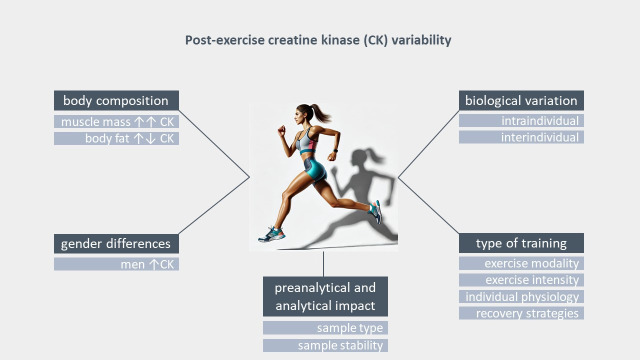

## Highlights

• High intensity exercise can induce muscle damage

• Monitoring creatine kinase (CK) activity can provide information about the extent of muscle damage

• CK variability following exercise is influenced by a range of factors

• Understanding variations is crucial for optimizing training and recovery strategies

## Introduction

Exercise-induced muscle damage (EIMD) refers to the structural disruption and physiological changes that occur within skeletal muscle fibers as a result of muscular contractions, unaccustomed exercise, eccentric contractions, or those under heavy loads (*1*, *2*). The commonly observed indicators of EIMD encompass increased serum activity of enzymes mostly specific for skeletal muscle damage, such as creatine kinase (CK), elevated inflammatory markers like interleukin (IL)-6, indications of oxidative stress such as hydrogen peroxide, delayed onset muscle soreness (DOMS), as well as prolonged impairment in functional performance (*3*-*5*).

Creatine kinase (CK) has been widely recognized as a reliable biomarker for assessing muscle function, damage, and recovery. However, the significance of elevated serum CK activity following physical exercise remains a subject of ongoing debate (*6*-*9*). It is well-established that the appearance of muscle-specific CK in the serum typically peaks 2-6 days after the initial insult, often coinciding with the onset of DOMS (*2*, *10*). Moreover, increased CK activity has been observed across a diverse range of exercise modalities, including high- and low-intensity activities, eccentric exercise, varying contraction velocities, and different rest intervals between sets (*11*-*22*). Notably, elevated CK activities have been associated with significant impairments in athletic performance and an increased risk of injury, highlighting the importance of understanding the factors that contribute to CK release and its implications for exercise training and recovery.

Noteworthy, while elevated CK activity is a well-established indicator of muscle damage, its interpretation is complex due to substantial inter-individual variability. Factors influencing this variability include contraction type (*e.g.*, eccentric *vs.* concentric), exercise intensity, and volume, as well as individual characteristics such as training status, age, and sex (*8*, *23*-*27*). Furthermore, the appearance and magnitude of the CK response can vary significantly between individuals, with some exhibiting minimal elevations despite substantial training loads (*8*).

**Figure 1:**
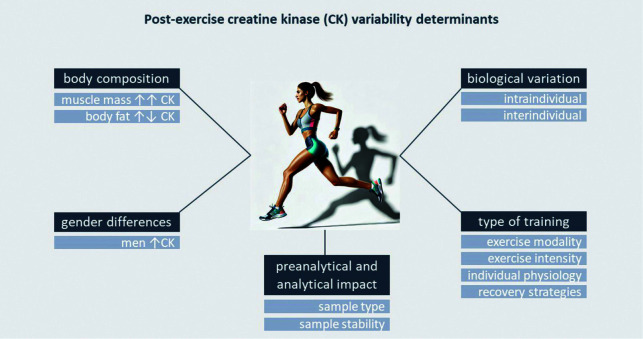
Post-exercise creatine kinase variability determinants. CK - creatine kinase.

Understanding the predictors of CK variability is paramount for optimizing training and recovery strategies in athletes. Furthermore, exploring these factors can significantly enhance evidence-based practices for preventing overuse injuries and promoting long-term athletic performance and health. Therefore, the aim of this literature review was to comprehensively examine the range of factors that contribute to variations in CK activities following different modalities of exercise, as well as to give detailed overview of the possible (pre)analytical factors that contribute to overall variability (Figure 1).

## Materials and methods

Two major electronic databases, Web of Science and PubMed were searched from inception to March 31, 2024, using the following search terms: variability AND (“creatine kinase” OR CK) AND (sport OR exercise OR acute). Two different authors (N.T. and V.R.B.) independently assessed the eligibility criteria of the selected studies. The following inclusion criteria were: (*1*) healthy, recreationally active and/or professional athletes without age and sex restrictions; (*2*) we considered both randomized and non-randomized studies published in peer-reviewed journals with a Journal Citation Reports Index; studies published in English; (*3*) studies examining the acute changes in CK (CK variability) were included in further analysis.

We excluded studies that met any of the following criteria: studies including clinical populations or participants diagnosed with any chronic disease (*i.e.*, cardiovascular diseases, cancer, diabetes mellitus type 2) and/or musculoskeletal injuries, and ineligible publication types (*e.g.* editorials, letters, commentaries, unpublished studies, guidelines).

Search Results revealed 981 results in PubMed, and 457 results in WoS database. The selection process was conducted in three steps. First, two authors (N.T. and V.R.B.) independently identified and flagged potentially eligible studies through extensive searches in each database. Second, after removing duplicates, potentially relevant studies were identified by reviewing titles and abstracts. Finally, the full text of articles that seemed eligible was reviewed by a third author (A.L.) to determine their suitability for further analysis.

## Post-exercise creatine kinase variability determinants

### Type of training and exercise intensity

Creatine kinase variability following different types of exercise is significantly influenced by both the type of training and exercise intensity. Specifically, resistance training, particularly involving eccentric contractions, induces substantial CK elevations up to 100% baseline value due to muscle fiber damage, with prolonged increases in CK observed following high-intensity or high-volume resistance exercise, ranging from 300-6000 IU/L (*7*). Eccentric contractions occur when a muscle lengthens while producing force. This type of contraction often happens when a muscle resists an external load or controls movement as it returns to its original length after a shortening (concentric) phase. Sports often involve eccentric contractions, such as landing from a jump or decelerating during running. Conversely, aerobic exercises, such as endurance running or endurance training, typically result in lower CK elevations (Table 1) (*28*). The benefits of endurance training include increased stamina, improved cardiovascular health, and enhanced metabolic efficiency. Endurance training involves engaging in physical activities designed to enhance the body’s ability to sustain prolonged exercise by improving cardiovascular and muscular endurance (*29*). Additionally, high-intensity interval training (HIIT), a workout method that alternates short intervals of intense physical activity with shorter periods of rest or low-intensity activity, also results in elevated CK activities, similar to resistance training (*30*). This method allows for effective training within a shorter period, promoting significant adaptations in the cardiovascular, metabolic, and muscular systems. The mechanisms underlying these increases involve intense stress on active muscles coupled with the activation of fast-twitch muscle fibers, also known as Type II fibers, specialized muscle cells designed for short bursts of powerful and rapid movements, which are more susceptible to damage. Conversely, aerobic training such as endurance running leads to smaller elevations in CK, which is expected given the predominant activation of slow-twitch muscle fibers during this type of exercise (*28*).

**Table 1:** Creatine kinase activity increase relative to type of training and exercise intensity

**Intensity**	**Aerobic exercise**	**Resistance exercise**	**Flexibility, balance,** **and other exercise**
Low intensity	**~**	↑	**~**
Example	Walking, light cycling and swimming	squats (slow pace), wall push-ups, resistance band exercises	Static stretching, dynamic stretching, Yoga, Tai Chi (movements to enhance flexibility)
Moderate intensity	↑	↑↑	~
Example	Jogging, cycling, skiing	Push-ups, moderate-weight dumbbell exercise	Pilates, Bosu ball exercises, stability ball exercises, Tai Chi (faster-paced movements with greater range of motion)
High intensity	↑↑	↑↑	↑
Example	Football match, HIIT, running sprints or vigorous cycling	Powerlifting-style exercises, heavy lifting	Dance
HIIT - high intensity interval training. ~ - low to none CK increase. ↑ - moderate increase. ↑↑ - high increase.			

Furthermore, CK variability following exercise is intricately linked to exercise intensity, with higher intensities generally eliciting greater CK elevations due to increased muscle damage. More precisely, high-intensity activities induce substantial CK increases reflecting the intense muscle stress and microtrauma (*29*). Conversely, moderate-intensity activities exhibit lower CK variability (*30*, *31*). Several individual studies corroborate this observation. For instance, Cerqueira *et al.* examined the inflammatory effects of exercise and observed that while both high and moderate intensities elicit CK increases, the magnitude is generally greater with higher intensity efforts (*30*). Furthermore, Paschalis *et al.* investigated the relationship between exercise intensity and muscle damage by comparing equal volumes of high and low-intensity eccentric exercise (*11*). Their findings revealed that even when total volume is controlled, higher intensity eccentric exercise induces more pronounced CK increases.

However, generalizing results solely based on training type and intensity is ill advised, as individual factors (*e.g.*, training status, muscle mass, and recovery strategies) significantly contribute to CK variability (*32*). Moreover, repeated bouts and type of contraction further complicate CK variability, as prior exposure to eccentric exercise can attenuate subsequent CK elevations, indicating an adaptive protective mechanism (*32*). The repeated bout effect refers to the phenomenon where a muscle becomes more resistant to damage and soreness after being exposed to an initial unaccustomed exercise. Essentially, the body adapts to protect the muscles from experiencing the same level of damage or soreness during subsequent workouts. The adaptation has been attributed to neural, connective tissue or cellular adaptations (*33*). In contrast, CK elevation and variability following single bouts of typical gym and fitness studio exercises reached up to 5.5 times baseline levels or 900 U/L (*32*).

Therefore, while training type and intensity influence CK variability, other training and individual characteristics significantly impact CK fluctuations. Consequently, understanding how these factors influence CK variability is paramount for optimizing training programs, enhancing recovery, and preventing overtraining and injury in athletes.

### Creatine kinase variability and gender differences

Creatine kinase variability is notably influenced by gender differences, with several studies highlighting the distinct responses of males and females to EIMD. Research by Hicks *et al.* demonstrated that following maximal eccentric knee extensions, males exhibited significantly higher CK activities compared to females, suggesting a greater degree of muscle damage in men under similar exercise conditions (*34*). This difference may be attributed to larger muscle mass and greater absolute strength in men, which could result in more substantial muscle fiber disruption during intense exercise (*35*).

The protective role of estrogen in women has been proposed as a key factor in these gender differences. Estrogen is known for its antioxidant properties and its ability to stabilize muscle cell membranes, potentially reducing the extent of muscle damage and the subsequent release of CK into the bloodstream (*36*). Oosthuyse and Bosch explored the effects of gender and menstrual phase on CK activity and muscle soreness following downhill running, finding that women generally had lower CK activity post-exercise compared to men (*37*). Specifically, they found that regardless of their menstrual phase, women demonstrated a faster return to baseline CK values within 48 hours post-exercise compared to men, who required 72 hours. Additionally, they observed that CK activity in women varied across the menstrual cycle, with different phases showing different levels of muscle damage and soreness. This suggests that hormonal fluctuations in women may further modulate CK responses to exercise, adding another layer of complexity to gender differences in CK variability. Danielsson *et al.* also reported gender differences in CK responses following an Ironman-distance triathlon, where male participants had higher post-race CK activities than females (*38*). This finding aligns with the general trend of greater CK elevations in men following strenuous exercise, reinforcing the idea that men are more prone to EIMD. However, it should be emphasized that females show a lower baseline CK activity (81 U/l) compared to males (139 U/l), but the females have a somewhat greater peak and relative increase in serum CK activity in response to the exercise (*39*). This was confirmed with Clarkson and Sayers stating that while men often show higher CK responses, women tend to experience prolonged muscle soreness despite lower CK activities, indicating that CK might not fully capture the extent of muscle damage or the recovery process in women (*40*). Understanding these differences is essential for developing gender-specific training and recovery protocols that account for the unique physiological responses of men and women.

### Inter-and intra-individual variability in creatine kinase

Individual characteristics also play crucial roles in determining CK variability. This can be attributed to a range of factors including fitness level, genetic predisposition, training history, and the specific nature of the exercise performed. Cipryan found that individuals with higher fitness levels exhibited a more controlled and less pronounced CK response following HIIT, likely due to enhanced muscle conditioning and better recovery mechanisms (*30*). This suggests that more trained individuals are better able to manage the muscle damage induced by intense exercise, resulting in lower CK elevations. The length of rest intervals between resistance exercise sets also plays a role in CK variability, as demonstrated by Silva *et al.* (*41*). Their study highlighted significant inter-individual differences in CK activity based on how rest intervals were structured. Some individuals showed marked increases in CK with shorter rest intervals, while others did not, indicating that individual recovery capacity and muscle resilience can greatly influence CK responses.

Creatine kinase variability is highly individualized, with factors such as fitness level, genetic predisposition, training history, and exercise types all contributing to the range of responses observed, as shown in some examples in Table 2.

**Table 2:** Examples of creatine kinase intra- and interindividual variability among athletes

**Study** **(reference)**	**Sample size, groups (N, M/F)**	**Characteristics of the study** **sample**			**Time points**	**CK (U/L)**	**Change in CK** **(CV%)**	**CK method and storage** **conditions**
		**Age (y)**	**Weight (kg)**	**Status**				
Nosaka *et al.* (*42*)	10 (M)	22 ± 2	64.7 ± 12.4	Non-trained students	Before, after, and 7 days after exercise sessions	12,873 ± 9679	75%	Serum samples stored at - 20°C until analysis. Spectrophotometrically (Dinabot Co. Ltd Japan)
Seifert *et al.* (*43*)	10 (F)	23 ± 4	N.A.	Recreationally active students	CK was measured after breakfast, after the 2^nd^, 12^th^ and 24^th^ ski run	Pre - 40.4 ± 19.3 Post - 57.3 ± 25.4	Pre - post 42% Individual CV% - 73%	Earlobe blood sample analyzed by reflectance photometry at 25°C for CK (Reflotron; Roche Diagnostics, Basel, Switzerland).
Hammouda *et al.* (*44*)	15 (M)	17 ± 0.3	69.1 ± 4.2	Professional football players	Before and 3 minutes after each exercise session	Day off: Morning - 160.45 ± 18.68 Evening - 147.9 ± 173.0 Exercise day: Morning - 191.18 ± 21.13 Evening - 219.27 ± 27.74	Day off: Morning - 11.6% Evening - 8.3% Exercise day: Morning - 11.1% Evening - 12.7	Creatine-kinase activity was determined spectrophotometrically on Synchron CX systems (Beckman Instruments, Danville, California, USA) by using Randox reagents.
Russel *et al.* (*45*)	14 (M)	Under 21	N.A.	Professional football players (U-21)	CK was measured at baseline pre-match, and 24h, and 48h after match	Pre - 343 ± 150 24 hours - 334.8 ± 107.2 48 hours - 156.9 ± 121.0	Pre - 41.7% 24h - 30.0% 48h - 34.3%	Storage at - 70°C before analysis on Cobas Mira (ABX Diagnostics, Northampton, United Kingdom).
Sayers and Clarkson (*46*)	25 (M) 25 matched controls	IMM: 20 ± 0.4 CON: 21± 0.6	IMM: 72.8 ± 3.3 CON: 75.0 ± 3.2	College males (nonweight-trained individuals)	Baseline and 9 days after exercise and immobilization	IMM: 955 +/- 316 IU/L *vs.* CON: 2884 +/- 1083 IU/L	There was little variability among participants in immobilization group	Serum samples stored at - 70°C until analysis. Measured by a Bausch & Lomb spectrophotometer and a Sigma Diagnostics kit (St. Louis, USA)
Becker *et al.* (*47*)	11 (M)	18 ± 1	72 ± 6	Male youth soccer players (U-19 first league)	Mon-Wed-Fri for three weeks at the beginning of each training session, two times and the midpoint and at the end of the half season	47 to 665, median 241 U/L at midpoint of the half season 54 to 791 median 212 U/L at the end of the half season	Intra-individual variability 50-58%, inter-individual variability 33-64%	Capillary blood from the earlobe, analyzed immediately on Reflotron Plus system (Roche Diagnostics, Manheim, Germany)
Gastin *et al.* (*48*)	26 (M)	23 ± 3	85.8 ± 7.4	Professional Australian football players	Baseline value before season, pre-match 24-36h before match, post-match 34-40h after match	Baseline: 73 ± 53 Pre-match: 354 ± 170 Post-match: 691 ± 345	Baseline: 72.6% Pre-match: 48% Post-match: 50%	Capillary blood from the fingerprick, analyzed immediately on Reflotron Plus system (Roche Diagnostics, North Ryde, Australia)
Wiig H *et al.* (*49*)	75 (M)	20 ± 5	72.7 ± 7.2	Outfield football players	Pre-match: 144, 72, and 1h pre-match Post-match: 1, 24, 48, and 72h after match	Baseline values: 367 ± 273 Post-match presented as effect size (SD/baseline value)	Baseline values: 74.4%	Serum samples stored at - 80°C until analysis. Analyzed on Cobas 8000 (Roche Diagnostics, Manheim, Germany).
Mendes *et al.* (*50*)	35 (M)	26 ± 5	79.1 ± 7.0	Professional soccer players	Sampling was performed before the training session	Variations of internal loads between particular playing positions: 238.7 ± 166.8	Average: 71.1%	Capillary blood from the fingerprick, analyzed immediately on Reflotron Plus system (Roche Diagnostics, Manheim, Germany)
Alaphilippe *et al.* (*51*)	12 (M)	21 ± 1	100.1 ± 11.4	High-level rugby players	Sampling was performed during the recovery session. The tests were longitudinally carried out every 15 days over the course of one sporting season (July to March).	The CK values were longitudinally presented on a graph, but not with absolute values and measures of uncertainty.	Average CK values were approximately 400 U/L, with a significant variation during the months (approximately from 100 to 600 U/L)	Capillary blood from the fingerprick, analyzed immediately on Reflotron Plus system (Roche Diagnostics, Manheim, Germany)
Obminski *et al.* (*52*)	13 (M)	25 ± 2	67.1 ± 13.2	Amateur boxers	Sampling was performed each morning during 16 consecutive days during early-season training	650 ± 297	45.7% (within-person variability ranged from 47% up to 78%)	Capillary blood from the earlobe. Serum samples stored at - 80°C until analysis. Analyzed at Biotechnica Instruments, Italy.
Proia *et al.* (*53*)	8 (M)	23 ± 2	78.3 ± 4.5	Professional soccer players	Basal values, and after three weeks of training	Basal: 429 ± 70 After: 233 ± 60	Before: 16.3% After: 25.7%	Venous blood samples, serum samples stored at - 80°C until analysis. Analyzed at Targa 3000, Biotecnica.
Skorski *et al.* (*56*)	161 (M)	N.A.	N.A.	Professional soccer players	Sampling was performed after a day off and 48 h after strenuous training	Day off: 265 ± 283 After training: 440 ± 308	107% 70%	Venous blood samples. Analysis method not mentioned in the manuscript.
Yamin *et al.* (*57*)	70 (28 F)	25 ± 3	67 ± 10	Physically active students	Pre, 3, 24, 48, 72, 96, 120, and 168h after exercise	Baseline: M: 119.9 ± 39.7 F: 109.9 ± 32.4 ΔCK: M: 4859.7 ± 6809.9 F: 3515.5 ± 4163.5	33.1% 29.5%	Whole blood creatine kinase activity from antecubital vein on Reflotron system.

In a study on recreationally active female students CK significantly increased during and after active skiing, however large variability occurred among individuals, although the study subjects belonged to the same gender and were very similar in terms of age (22 ± 4 years) (*43*). Although CK can be a promising biomarker for internal load estimation when assessed frequently, Gastin and Becker concluded that CK as a global measure of muscle damage can be used with caution given the high intra- and inter-individual variability, with establish intra-individual variability as high as 50-58% on a group of male youth soccer players (*47*, *48*). Large variations were also observed in a study by Wig, also in a group of football players, where baseline values varied up to 75% between teammates (*49*). Obminski *et al.* who studied a group of young amateur boxers found somewhat lower within-person variability, 46% on average, however their fluctuations were highly variable during early-season training (*52*). Perhaps that can be connected to training adaptations during the season, with an observed decrease in CK variability after 3 weeks of training, as observed by Proia *et al.*, Ribeiro *et al.*, and Alves *et al.* (*53*-*55*). All emphasized the importance of establishing individual-based CK reference values, as players in team sports exhibited wide-ranging CK responses to similar workloads. This is in accordance with studies by Skorski *et al.* and Yamin *et al.* who underlined that CK variability cannot be predicted by age, race, body composition and physical activity (*56*, *57*). They propose assessing muscle recovery by using individualized ranges that seem to offer a higher diagnostic accuracy than a sample-specific group-based analysis. Recognizing and accounting for these individual differences is crucial for optimizing training and recovery protocols, as well as for accurately interpreting CK activities as a marker of muscle damage and recovery in both athletic and clinical settings.

### Body composition

Variability in CK activity is strongly influenced by body composition, with evidence suggesting that muscle mass, fat content, and genetic factors substantially influence CK activities post-exercise. Kim and Lee found a strong relationship between CK variability and body composition, particularly after eccentric muscle contractions (*23*). Their study demonstrated that individuals with greater muscle mass tended to have higher CK activities post-exercise, likely due to the larger volume of muscle fibers that can be damaged during intense physical activity. This suggests that athletes with more muscle mass may experience greater CK elevations as a natural consequence of their physique. Regarding body mass index (BMI), Kim *et al.* found that high BMI group showed higher CK activities at 24, 48, 72, and 96 h after exercise when compared to the findings in the normal BMI group (*58*).

Conversely, body fat percentage also plays a role in CK variability. Bekkelund and Jorde observed that higher body fat levels were associated with increased CK responses in an overweight and obese population (*59*). They proposed that the elevated CK activities could be linked to a higher proportion of type II muscle fibers in individuals with obesity, which are more susceptible to damage and consequently lead to greater CK release. Similarly, Kim and Lee found that individuals with higher body fat percentages exhibited greater variability in CK activities following eccentric exercise (*23*). Their results indicated that increased adiposity was associated with a higher inflammatory response and greater muscle damage, as reflected by elevated CK and other muscle damage markers. Although the exact mechanisms remain complex, these studies suggest that excess body fat does not provide a protective cushioning effect during exercise.

Further, the Tromsø Study, as reported by Lilleng *et al.*, highlighted the natural variability in serum CK activities across a normal population, emphasizing that even within a generally healthy group, CK can vary widely due to differences in body composition (*60*). Persistent hyperCKemia, or chronically elevated CK activities, was more commonly observed in individuals with greater muscle mass, further supporting the link between muscle size and CK variability.

Additionally, genetic factors such as specific genotypes related to creatine kinase and angiotensin-converting enzyme (ACE) can influence how body composition affects CK responses. Heled *et al.* identified that certain genotypes were predictive of higher CK responses to exercise, suggesting that genetic predispositions, combined with body composition, create a unique CK response profile for each individual (*61*).

Creatine kinase variability is closely linked to body composition, with muscle mass generally leading to higher CK activities post-exercise, while higher body fat may correlate with lower CK responses (Figure 1).

## Preanalytical and analytical considerations

The active form of CK (EC 2.7.3.2) is a dimer composed of two subunits (M and B) resulting in three different isoenzymes numbered based on their electrophoretic mobility: BB (or CK-1), MB (or CK-2) and MM (or CK-3). They are found in the cell’s cytosol or are associated with myofibrillar structures. Their presence differs among various tissues: skeletal muscle (CK-MM), heart (CK-MM and small amount of CK-MB), brain (CK-BB), gastrointestinal tract and urinary bladder smooth muscle (CK-BB). The measurement of plasma enzyme activity is important in the diagnosis of muscle disease, and while the activities of several enzymes may be elevated, CK is the most sensitive indicator of muscle damage. Skeletal muscle has the highest CK content of any tissue, more than three times as much as heart or brain, and consequently nearly all CK activity in normal plasma is derived from skeletal muscle (*62*, *63*).

Although there are several methods available for total CK measurement, including photometric, fluorometric and coupled enzyme methods, a great improvement in standardization was obtained after International Federation of Clinical Laboratory Medicine (IFCC) established a reference method based on specific reaction principles for measuring CK at 37 ºC (Table 3) (*64*).

**Table 3:** International Federation of Clinical Laboratory Medicine reference method for creatine kinase measurement

creatine phosphate + ADP → CK → creatine + ATP
ATP + glucose → HK → glucose-6-phosphate + ADP
glucose-6-phosphate + NADP^+^ → G6PD → 6-Phosphogluconate + NADPH + H^+^
CK - creatine kinase. HK - hexokinase. G6PD - glucose-6-phosphate dehydrogenase. Adapted from (64).

The reaction mixture for CK measurement should contain several important components that play key roles in ensuring reliable measurement: N-acetylcysteine activates CK, ethylenediaminetetraacetic acid (EDTA) binds Ca^2+^ ions and increases stability of the reaction mixture, and adenosine pentaphosphate along with adenosine 5’-diphosphate (ADP) inhibits adenylate kinase. The production of NADPH, measured at 340 nm is proportional to the activity of present CK (*64*). Despite it took years for manufacturers and laboratories to implement the standardized method, to this day, CK measurements are very well harmonized (*65*-*67*). However, due to some observed differences in catalytic activity of CK prior and post standardization, one must always be careful when comparing literature data with a substantial year gap in-between.

As seen in Table 2, there is also a substantial variety in commonly used samples for CK determination in some studies, which include serum, plasma and sometimes capillary whole blood. Heparin is the only accepted anticoagulant in collection tubes, as other anticoagulants inhibit CK activity (*63*). Knoblauch *et al.* showed that capillary and venous samples are similar for total CK measurements after eccentric exercise, thus enabling using easier alternate capillary sampling technique to be used in the studies (*68*). The applicability of dry-reagent analyzers, such as Reflotron systems (Roche Diagnostics, Rotkreuz, Switzerland) widely used in many studies, became an option even in the 90s. In a large European study, fresh samples of human blood, plasma and serum were examined by Reflotron Plus System (Roche Diagnostics, Rotkreuz, Switzerland) CK and by an N-acetylcysteine activated creatine kinase method in six different clinical laboratories. The correlation between these methods was excellent (correlation coefficient ≥ 0.99), the median systematic deviation (bias) for all samples being smaller than - 5%. It was considered as an suitable alternative for decentralized testing sites, especially where CK results were needed quickly (*69*). The greatest challenge in CK measurement is the sample stability. Literature data vary, depending on the storage temperature (Table 4).

**Table 4:** An overview of literature data on creatine kinase sample stability

**Sample type**	**Storage temperature (°C)**				**Reference**
	**20 - 25**	**2-8**	**< (- 20)**	**< (- 70)**	
Serum	12 hours	3 days	4 weeks	NA	(*70*)
Serum	Relatively unstable if no protective agents were added	NA	NA	NA	(*63*)
Serum	4 hours	8 - 12 hours	NA	NA	(*71*)
Serum	CK very unstable, especially after 7 days	/	unstable	90 days	(*72*)
Plasma/serum	NA	7 days	NA	NA	(*73*)
	NA	NA	Unstable if no protective agents were added	Relatively stable	(*74*)
Serum	8 hours	48 hours	1 month	Longer stability	(*75*)
CK - creatine kinase. NA - not available.					

Perhaps, if the samples could not be analyzed as soon as possible, the most suitable storage conditions would be at or below - 80 °C, where CK was shown to be stable for at least 90 days (*72*). A more comprehensive overview of sample stability is given in Table 4. These limitations should always be taken into consideration when analyzing CK activity in stored samples for research or clinical purposes.

## Laboratory considerations for measuring creatine kinase activity in healthy, physically active non-athletes

Clinical interpretation of laboratory abnormalities after acute exercise often does not reflect the onset of pathology. In trained, and especially in untrained individuals, acute exercise can result in elevated serum enzyme activities that may last for hours to days. Nevertheless, caution is necessary in their interpretation because the reference ranges differ between sedentary populations, recreational athletes, and especially elite athletes. The pattern of change in CK differs in trained *versus* untrained individuals (*76*). Resting CK values are higher in trained individuals, while in untrained individuals, CK increases progressively for up to 5 days after acute exercise. The peak values measured can be 33-fold the baseline values (*77*). Laboratory implications for CK measurement in healthy, physically active non-athletes must be recognized and, to avoid misinterpretation, every vigorous exercise prior to blood sampling should be recorded.

## Limitations

Several limitations must be acknowledged to provide a balanced and accurate interpretation of the findings. This mini-review, provides a concise overview but may not capture the full depth of the existing literature. As a result, some important studies or nuanced findings related to CK variability could be overlooked, leading to a less comprehensive understanding of the topic. A systematic review could provide stronger evidence regarding CK variability. Moreover, there is a great variability in methodological approaches, differences in study designs, such as the type of exercise protocols used, the timing of CK measurements, and the methods of CK analysis, which can introduce variability in the results. This makes it difficult to directly compare findings across studies, potentially leading to inconsistent conclusions. Acknowledging these limitations is crucial for understanding the scope and applicability of the findings, as well as for guiding future research that can address these gaps and provide a more comprehensive understanding of CK variability in response to different factors.

## Future directions

Further research into CK variability should explore several key areas to deepen our understanding and address existing gaps. Future research should focus on long-term studies that track CK variability over extended periods, particularly in relation to chronic training adaptations. This would help in understanding how CK activities fluctuate not just in the short term but also across different phases of training cycle. Research should explore how individual differences, such as body composition, fitness levels, and genetic predispositions, can be used to develop personalized recovery strategies that optimize CK activities and overall muscle recovery. This could lead to more effective, individualized approaches in sports medicine. Future research could also explore the development and validation of non-invasive or minimally invasive methods for monitoring CK activities, as shown by Kucherenko *et al*. (*78*). Such technologies could provide real-time feedback to athletes and coaches, enhancing the ability to monitor muscle recovery and prevent overtraining. By addressing these areas, future research can provide deeper insights into CK variability, leading to improved training, recovery strategies, and overall athlete performance.

## Conclusions

Creatine kinase variability following exercise is influenced by a range of factors, including the type and intensity of exercise, individual differences such as genetic predispositions and body composition, and gender-specific responses. Understanding these variations is crucial for optimizing training and recovery strategies, as well as for preventing muscle damage and overtraining. This review article uniquely describes the possible variations in CK measurements after acute exercise, emphasizing preanalytical and analytical considerations that contribute to the variability in general, and are often overlooked. By connecting sports medicine and laboratory medicine into a single entity, greater utility is possible in the care of both professional and recreational athletes, whose laboratory findings may also be changed due to an active lifestyle. Continued research into CK variability will enhance our ability to tailor exercise and recovery protocols to individual needs, ultimately improving athletic performance and reducing injury risk.

## Data Availability

No data was generated during this study, so data sharing statement is not applicable to this article.
